# Strategies for Improving Ocular Drug Bioavailability and Corneal Wound Healing with Chitosan-Based Delivery Systems

**DOI:** 10.3390/polym10111221

**Published:** 2018-11-03

**Authors:** Teodora Irimia, Mihaela Violeta Ghica, Lăcrămioara Popa, Valentina Anuţa, Andreea-Letiţia Arsene, Cristina-Elena Dinu-Pîrvu

**Affiliations:** 1 Department of Physical and Colloidal Chemistry, Faculty of Pharmacy, University of Medicine and Pharmacy “Carol Davila”, Bucharest 020956, Romania; teodora.irimia@drd.umfcd.ro (T.I.); mihaela.ghica@umfcd.ro (M.V.G.); valentina.anuta@umfcd.ro (V.A.); cristina.dinu@umfcd.ro (C.-E.D.-P.); 2 Department of Pharmaceutical Microbiology, Faculty of Pharmacy, University of Medicine and Pharmacy “Carol Davila”, Bucharest 020956, Romania; andreeanitulescu@hotmail.com

**Keywords:** chitosan, chemical modification, physical gels, ocular drug delivery, corneal wound healing

## Abstract

The main inconvenience of conventional eye drops is the rapid washout of the drugs due to nasolacrimal drainage or ophthalmic barriers. The ocular drug bioavailability can be improved by either prolonging retention time in the cul-de-sac or by increasing the ocular permeability. The focus of this review is to highlight some chitosan-based drug delivery approaches that proved to have good clinical efficacy and high potential for use in ophthalmology. They are exemplified by recent studies exploring in-depth the techniques and mechanisms in order to improve ocular bioavailability of the active substances. Used alone or in combination with other compounds with synergistic action, chitosan enables ocular retention time and corneal permeability. Associated with other stimuli-responsive polymers, it enhances the mechanical strength of the gels. Chitosan and its derivatives increase drug permeability through the cornea by temporarily opening tight junctions between epithelial cells. Different types of chitosan-based colloidal systems have the potential to overcome the ocular barriers without disturbing the vision process. Chitosan also plays a key role in improving corneal wound healing by stimulating the migration of keratinocytes when it is used alone or in combination with other compounds with synergistic action.

## 1. Introduction

The major difficulty in treating ocular diseases is to provide and maintain an optimal ocular concentration of the drug for a long period of time [1]. Topical instillation is by far the preferred route of administration in the treatment of anterior segment diseases. Ophthalmic drops are the most commonly-used formulations due to the ease of administration and patient compliance. However, the bioavailability of the active substance is less than 5% due to anatomical and physiological constraints such as lacrimal turnover, blinking reflex, nasolacrimal drainage or ocular barriers [2]. Only a very small volume of lacrimal fluid (7–9 μL) is physiologically found on the eye surface, while a droplet bottle releases a higher volume of fluid [3]. When the volume of a drop exceeds 30 μL, nasolacrimal drainage and gravity lead to the loss of the drug [4]. Furthermore, the high surface tension of an aqueous drop prevents spreading on the ocular surface. Another disadvantage of the aqueous solutions is that since most of the chemical entities are lipophilic or high molecular weight compounds, their solubilization requires the addition of oils or surfactants that disturb the vision process and decrease the patient’s tolerability [5].

The first structural barrier of the eye is represented by the cornea, a transparent, avascular tissue. It consists of 5 layers: 3 cellular (epithelium, stroma, endothelium) and 2 interfaces (Bowman membrane, Descemet membrane) [6]. The epithelium and endothelium of the cornea have a high lipid content, which restricts the permeability of polar, water-soluble compounds. The stroma, which is a hydrophilic layer, is made up of 70–80% water, and it represents a barrier to liposoluble compounds [7].

Melanin binding affects the pharmacokinetics of the drugs. Topically-applied substances permeate the cornea to aqueous humor, reaching melanin-containing tissues such as iris and the ciliary body. Melanin binding is associated with increased retention in the pigmented tissues, leading to a prolonged response of therapeutics [8].

A certain fraction of the drug reaches the systemic circulation via conjunctival vessels and nasolacrimal duct [9]. From the anterior segment, the drug is removed by two ways: the aqueous humor turnover and the blood flow of the uvea. In the case of vitreous humor, elimination takes place in the anterior or posterior chamber, through the blood-retinal barrier [3].

**Figure 1:**
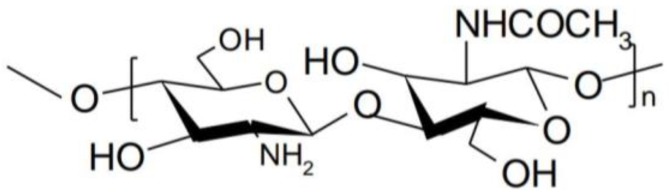
Structure of chitosan.

Chitosan is a polymer obtained by alkaline or enzymatic deacetylation of chitin, which is the main component in the exoskeleton of marine organisms such as crab, lobster, squid, shrimp, insect cuticle, the cell wall of some fungi and other organisms such as algae or yeasts. [10]. Chemically, chitosan consists of repeating units of *N*-acetyl-d-glucosamine and d-glucosamine linked by β-(1-4) glycosidic bonds (Figure 1) [11].

Chitin and chitosan possess interesting biological properties, so they have numerous applications especially in the medical and pharmaceutical fields. Chitin has low utilization compared to chitosan because it is insoluble in water and chemically unreactive. For this purpose, chemical modification of chitosan and chitin has been used to increase solubility and extend the spectrum of application [12]. Chitosan derivatives are obtained by chemical modification using techniques such as acylation, alkylation, hydroxylation, quaternization or esterification and have properties superior to unmodified chitosan [13].

Chitosan is a natural polymer and an inexpensive biomaterial, being an attractive excipient in the pharmaceutical industry by including it in various formulations such as prolonged or controlled drug delivery systems, wound dressings, hemostatic sponges, tissue engineering scaffolds or space filling implants [14].

Chitosan is a suitable candidate in ophthalmic formulations due to its biocompatibility, biodegradability, non-toxicity, mucoadhesive character, antibacterial and antifungal effects [15,16,17]. Its solutions have viscoelastic and pseudoplastic properties that do not disturb the tear film. Chitosan increases the permeability of the mucosal barriers and promotes wound healing [18]. It plays a key role in improving corneal wound healing by enhancing keratinocyte migration, which leads to rapid increase in collagen synthesis [19]. Cui et al. have shown that chitosan promotes the proliferation of the corneal epithelium during healing of an injury on the rabbit eye. Corneal wound healing is mediated by the activation of the extracellular signal-regulated kinases (ERK), a subfamily of mitogen-activated protein kinase (MAPK) [20].

The main purpose in the development of ophthalmic formulations is to obtain an optimal concentration of the active substance and to maintain it for a longer period of time, thus reducing the frequency of administration [21]. To overcome the inconvenience of topical ophthalmic preparations, researchers have approached two strategies:

1. Increasing corneal residence time using viscosity enhancers, mucoadhesive agents and in situ gels;
2. Increasing corneal permeability using penetration enhancers, prodrugs and colloidal systems such as nanoparticles and liposomes [22].

In this review, we highlight some chitosan-based drug delivery systems that proved to have good clinical efficacy and high potential for use in ophthalmology. They are exemplified by recent studies exploring in-depth the techniques and mechanisms in order to improve ocular bioavailability of the active substances. Some research works are developed by exploiting the potential of chitosan in promoting corneal wound healing when used alone or in combination with other compounds with synergistic action.

## 2. Strategies to Increase Residence Time on the Ocular Surface

### 2.1. Viscosity Enhancers

Some ocular drug delivery systems aim to achieve prolonged retention with reduced frequency of administration. Slightly viscous solutions are recognized as having better patient compliance. Increasing the viscosity of a preparation influences drug bioavailability by improving ocular retention time. It seems to be a tight range of viscosity between 15 and 18 centipoises (cPo), because the product must have negligible visual effects. Furthermore, it should be filterable and sterilized [23]. Tears have a viscosity of 1.5 mPa·s, but non-Newtonian flow due to the presence of mucin and other macromolecules. Studies have shown that a viscosity below 10 mPa·s leads to undetectable changes in drainage rate and does not affect ocular retention time [24].

Natural and synthetic polymers have been shown to be useful in ophthalmic formulations due to viscosity-increasing properties [25]. These polymers added to ophthalmic solutions result in a slow elimination rate from the ocular area. Often, cellulose derivatives (methylcellulose, hydroxypropyl methylcellulose, hydroxyethyl cellulose), poly(vinyl alcohol) (PVA) and poly(vinylpyrrolidone) (PVP) [26] are used. The addition of such polymers into ophthalmic formulations requires adequate technological solutions, as they may cause blurred vision due to changes in corneal refraction index or difficulty in instillation of a precise dose [25].

Hydrogels made up of chitosan alone show poor mechanical strength, low elasticity due to intrinsic rigidity of the chains and lack an efficient control of drug delivery. The addition of other polymers leads to the formation of polyelectrolyte complexes (PEC) with increased mechanical properties while maintaining all the properties of chitosan. PECs are preferable to chemical hydrogels, as there is no need for the addition of catalysts [27]. Hydrogels can be formed by polymer mixtures of chitosan with other non-ionic polymers such as poly(vinyl alcohol) (PVA) [28]. The combination of the two polymers is based on characteristics such as biocompatibility, biodegradability, non-toxicity and water solubility [29]. The gelling process of PVA determines the formation of a porous network in which junction points are represented by crystallites. Structural organization is described by a relative arrangement of chains in fringed micelle-like crystals and interactions between neighboring chains. Studies have confirmed that cross-linking of the gel occurs under the action of crystallites [30]. In the case of chitosan-PVA mixtures, increasing chitosan concentration negatively affects the formation of PVA crystallites by forming hydrogels with less ordered structures [28]. A study assessed the in vitro release of in situ gels based on Poloxamer 407 and chitosan. The addition of PVA (0.2%–0.3%) significantly prolonged ocular retention. According to the authors, the release rate of the system containing PVA was higher compared to that of the PVA-free system. The formulation exhibited a pseudoplastic behavior at 35 °C. The authors suggested that the association consisting of 20% poloxamer, 0.2% chitosan and 0.3% PVA (*w*/*w*) is a suitable formulation for ocular delivery of ciprofloxacin [31]. The development of chitosan-based biomaterials has potential for many applications due to its minimal foreign body sensation and intrinsic antibacterial properties. The field of tissue engineering allows the development of artificial cornea that adheres to the native cornea. Thus, it is necessary to create a scaffold with mechanical and transparent properties similar to the natural cornea. Seyed and Vijayaraghavan prepared and analyzed the properties of a PVA/chitosan-based scaffold cross-linked with 1-ethyl-3-(3-dimethylaminopropyl)-carbodiimide (EDC) and 2 *N*-hydroxysuccinimide (NHS) for in vitro delivery of cultured corneal epithelial cells. The viscosity of the solutions was measured using the Brookfield viscometer. For a 90:10 chitosan/PVA mixture, the viscosity value was 1726 cPo. A linear increase in viscosity for PVA solutions was also observed after the addition of increasing amounts of chitosan. Thus, blending chitosan with PVA solutions has the effect of thickening and increasing viscosity. The amino and hydroxyl groups of chitosan form hydrogen bonds with the PVA groups, leading to the formation of uniform nanofibers even at low PVA concentrations [32].

Cellulose derivatives are used in liquid formulations as viscosity enhancers. They are pH- sensitive polymers and have active surface properties that influence blinking rate [33]. Ahmed et al. incorporated polylactide-co-glycolide (PLGA) nanoparticles into polymeric gels in order to release ketoconazole. Hydroxypropyl methylcellulose (HPMC) was added to all preparations as a viscosity enhancer. The results showed that the addition of HPMC to chitosan-based formulations increased the viscosity following contact with simulated lacrimal fluid [34]. Silva et al. incorporated nanoparticles of chitosan, sodium tripolyphosphate and hyaluronic acid into a polymeric HPMC solution for ocular delivery of ceftazidime. The HPMC gel entrapped ceftazidime molecules, leading to prolonged release of the active substance. The diffusion rate of ceftazidime from nanoparticles could be modulated by varying vehicle viscosity. The drug diffusion through HPMC gel was lower than that of the nanoparticles alone because the enhanced gel viscosity produced a more compact polymeric matrix that decreased the diffusion rate of the active substance. HPMC does not have ionized groups to interact with the sialic acid residues of mucin. Therefore, HPMC’s ability to increase the drug retention time in the eye is more related to its capacity to enhance viscosity rather than to any interactions with mucus [35].

**Figure 2:**
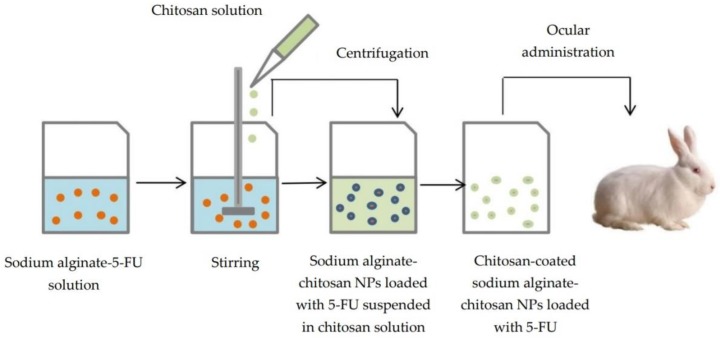
Preparation of chitosan-coated sodium alginate-chitosan nanoparticles with 5-fluorouracil (5-FU).

Due to the viscosity-enhancing effect, chitosan solutions enable the ocular retention of nanoparticles [36]. Chitosan-coated sodium alginate-chitosan nanoparticles with 5-fluorouracil (5-FU) were prepared by the ionic gelation technique and then suspended in chitosan solution, which is responsible for the enhanced viscosity, as nanoparticles did not show any interaction with mucin. In vivo study on rabbit eye showed a higher level of 5-FU in aqueous humor compared to 5-FU solution (Figure 2). The alginate shell was obtained by cross-linking with chitosan, which took place spontaneously through electrostatic interactions between negatively-charged carboxyl groups of alginate and protonated amino groups of chitosan [37].

### 2.2. Mucoadhesive Agents

Mucoadhesive delivery systems have the advantage of adhering to the mucous membrane layer. Mucosal adhesion increases the retention time of drugs and protects small, vulnerable molecules. The overall effects result in a controlled release of drugs with improved bioavailability and better patient compliance [38].

**Figure 3:**
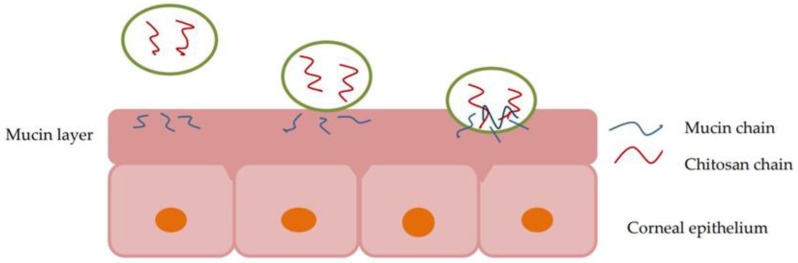
Interactions between chitosan chains and the mucin layer.

The mucinous layer keeps the film on the eye surface and creates an unfavorable environment for the growth of pathogens. Other important roles are to maintain the ocular surface moisturized, an optimal refractive index at the air-water-cornea interface and to keep solubilized proteins in tears [39]. Mucin is negatively charged at physiological pH (7.4) due to sialic acid residues from the terminal ends of the mucopolysaccharide chain, resulting in a preferential intake for cationic molecules. The use of positively-charged formulations is the most common way to increase the bioavailability of ocular formulations [7]. Chitosan has an attractive potential in improving the permeability through the mucosal epithelium due to its cationic structure and mucoadhesive character [40]. At low pH, the amino groups are protonated, and the polymeric macromolecules become positively charged. The cationic structure determines electrostatic interactions with the negatively-charged groups of sialic acid from epithelial surfaces (Figure 3). The mucoadhesive character is also influenced by hydrogen bonds and hydrophobic interactions [6].

The mucoadhesive mechanism implies two different phases: the contact phase and the consolidation phase. The first one involves the intimate contact between the mucoadhesive agent and the mucus, leading to the spreading and swelling of the formulation. In the consolidation phase, the mucoadhesive agent is activated by the presence of moisture, which allows the molecules to break free and to link up by van der Waals forces and hydrogen bonds. According to diffusion theory, mucoadhesive molecules interact with glycoproteins from mucus, forming secondary bonds [40].

In a study initiated by Yamaguchi et al., the effects of chitosan coating of an ophthalmic emulsion with indomethacin were analyzed. The authors evaluated the degree of retention of the chitosan-coated emulsion in lacrimal fluid and compared the results with those of an uncoated emulsion. The study showed a prolonged retention time of indomethacin in lacrimal fluid for the coated emulsion. The detachment force of coated chitosan emulsion from mucin was considerably higher than that generated by an HPMC emulsion with a similar viscosity, as a result of the mucoadhesive character of chitosan. According to researchers, the chitosan-coated emulsion proved to be a promising formulation for enhancing the bioavailability of indomethacin due to its adhesion to the ocular surface [41].

Li et al. attempted to develop a controlled delivery system for betaxolol hydrochloride useful in the treatment of glaucoma. They interlaced an active substance into the space between the layers of montmorillonite, which was then included in chitosan nanoparticles. The release of betaxolol from the solution was faster than that from nanoparticles. The drug remained detectable at the ocular surface for up to 12 min following application of the nanoparticles. This was attributed to increased mucoadhesive properties of chitosan nanoparticles. Mucoadhesive properties were based on interactions between the drug and the ocular surface, avoiding the rapid elimination of betaxolol. This fact determined an improved ocular bioavailability and better patient compliance [42].

Like chitosan, sodium alginate is a mucoadhesive polysaccharide. Sodium alginate possesses carboxyl groups that are partially negatively charged at neutral pH [43]. Shinde et al. developed mucoadhesive microspheres of chitosan and sodium alginate by the ionic gelation method in which they incorporated azelastine hydrochloride. For the chitosan-alginate complex, mucoadhesion is the result of a synergic action of these polymers that individually have mucoadhesive properties. In vitro results demonstrated that microspheres increased the retention time of azelastine due to the mucoadhesiveness of the system [44].

**Figure 4:**
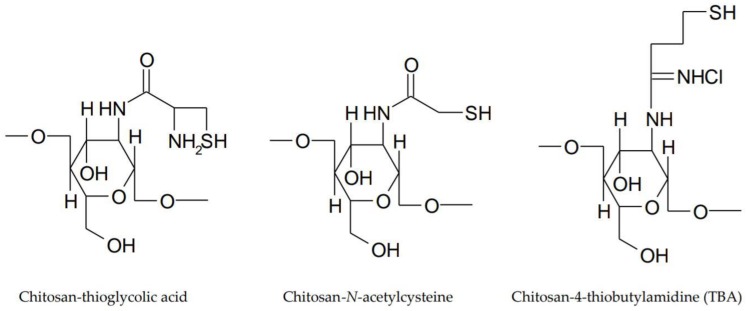
Thiolated derivatives of chitosan.

Substitution of the primary amino groups from the chitosan structure with thiol groups generates thiolated derivatives [45]. Three thiolated derivatives: chitosan conjugates with thioglycolic acid, chitosan-cysteine conjugates and chitosan-4-thiobutylamidine (TBA) conjugates were synthesized (Figure 4). The mucoadhesive properties of the thiolated derivatives are due to covalent bonds between the thiol groups of the polymer and the cysteine of the mucosal layer. Chitosan-thioglycolic acid conjugates have a 5–10-fold higher mucoadhesiveness compared to unmodified chitosan. In the case of chitosan-TBA conjugates, they have enhanced mucoadhesive properties due to ionic interactions between the additional amidine structure and the anionic mucosal layer. Studies have shown that average molecular weight conjugates have the highest mucoadhesive character [46].

Chitosan-*N*-acetylcysteine, a chitosan thiolated derivative, was approved as eye drops under the name Lacrimera^®^. Besides the good lubricating effect, it has two useful advantages in corneal wound healing. The introduction of thiol groups enables polymer interaction with mucin from the ocular surface [47]. Chitosan nanoparticles can rapidly disintegrate if they do not include compounds such as alginate that stabilize the system through an ion-cross-linking process. Thiolated chitosan nanoparticles do not disintegrate because disulfide bonds formed in the polymeric network stabilize the system and provide controlled drug delivery. Due to the immobilization of chitosan thiol groups, the addition of anionic compounds to thiolated chitosan nanoparticles leads to increased mucoadhesive properties [45]. Zhu et al. prepared nanoparticles based on chitosan, thiolated chitosan and sodium alginate as a potential ocular drug delivery system. Mucoadhesion studies showed that there is an increase in the number of electrostatic interactions between positively-charged nanoparticles and the negatively-charged mucosal surface. The addition of mucin reduced the zeta potential of nanoparticles. The electrostatic interactions between mucin and positively-charged amino groups were more pronounced in the case of thiolated chitosan-sodium alginate nanoparticles compared to chitosan-sodium alginate nanoparticles. Disulfide bonds promoted mucoadhesive properties. This fact made nanoparticles of thiolated chitosan-sodium alginate a more versatile delivery system, which fulfilled the requirements for application in the ophthalmic field [48].

**Figure 5:**
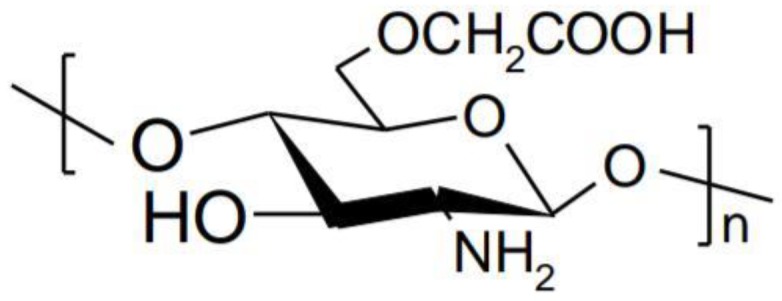
Chemical structure of *O*-carboxymethyl chitosan.

Carboxymethylation of chitosan generates soluble derivatives in both acidic and alkaline medium. These derivatives exhibit high viscosity, low toxicity, superior mucoadhesive properties and the ability to form gels [49]. Carboxymethyl chitosan is obtained by carboxymethylation of hydroxyl groups of chitosan. Reactive groups such as the carboxyl or amino group remain available for chemical modification in order to improve physical properties (Figure 5) [50].

Shinde et al. prepared 6-*O*-carboxymethyl chitosan from chitosan and monochloroacetic acid. The 6-*O*-carboxymethyl chitosan nanoparticles were developed by the ionic gelation method in order to provide a sustained release of dorzolamide in the eye. Mucoadhesion was evaluated in vitro by measuring the mucin binding efficiency of nanoparticles. The nanoparticles were spontaneously adsorbed to the mucinous layer due to the electrostatic attraction between the positive amino groups of 6-*O*-carboxymethyl chitosan and the negative sialic acid groups. In addition to ionic interactions, hydrogen bonds were present as a consequence of hydrophilic carboxyl groups. After incorporation of dorzolamide, the mucoadhesion of nanoparticles was lower. This was explained by the increase in particle size, which reduced the adsorption on the mucin surface [51].

Hyaluronic acid increases precorneal retention time because it has viscous and mucoadhesive properties. It also has other beneficial effects on the corneal epithelium, including protection against dehydration, reduction of healing time, reduction of inflammation response caused by dehydration and lubrication of the ocular surface [52]. Xu et al. formulated a mixture of carboxymethyl chitosan, hyaluronic acid and gelatin as the corneal epithelial cell carrier for corneal wound healing. Carboxymethyl chitosan, which is a water-soluble derivative, has wound healing properties. Gelatin facilitates cell adhesion and proliferation during healing processes. The results showed that a large number of corneal epithelial cells were attached to each other. The cells maintained normal morphology and adhesive activity. At the end of the study, the authors observed a complete repair of the rabbit eye area with corneal damage induced by alkali [53].

### 2.3. Ocular In Situ Gels

Ocular in situ gels are systems that are applied as solutions or suspensions and undergo a rapid sol-gel transition triggered by external stimuli such as temperature, pH or ionic strength, after instillation [54]. These formulations also combine the advantages of solutions such as accuracy and reproducibility of dosing, ease of administration, minimum interference with vision (absence of blurred vision, lack of sticking eyelids) with prolonged corneal residence, resistance to nasolacrimal drainage, reduced frequency of administration and characteristics of ointments [55]. These ophthalmic forms containing polymers are liquid at room temperature (25 °C) and undergo sol-gel transition upon contact with the ocular surface [56]. Depending on the physiological mechanisms that produce the gelation of polymers, there are three major categories of polymers:

1. Temperature-triggered in situ gelling polymers. The phase transition temperature is called the low critical system temperature (LCST). Below this value, the hydrogen bonds between the hydrophilic groups of the polymer and the water molecules improve dissolution of the polymer, and the system is a solution. As the temperature rises, the hydrogen bonds break, hydrophobic interactions appear and sol-gel transition takes place [57].
2. pH-triggered in situ gelling polymers. pH-responsive polymers contain weak acidic or basic groups that release or accept protons in response to pH changes. Thus, conformational changes occur in the polymer structure that determine its swelling.
3. Ion-triggered in situ gelling polymers. Cross-linking of sensitive polymers takes place due to monovalent or divalent cations in the tear film [58].

**Figure 6:**
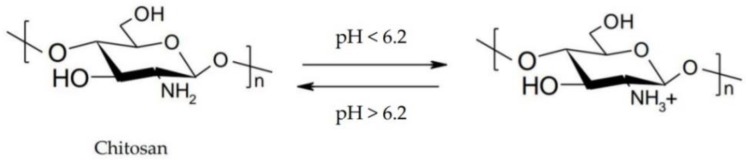
Schematic illustration of the protonation-deprotonation balance of chitosan.

Chitosan is a pH-responsive polymer, which remains in aqueous solution up to 6.2 [59]. It has pH-sensitive properties due to the protonation-deprotonation balance of amino groups, which allows the formulation of controlled release systems (Figure 6) [60].

Chitosan is able to transform into an ocular gel at pH 7.4. Secondary chemical linkages such as hydrogen bonds or ionic interactions appear between positively-charged amino groups and negatively-charged sialic acid residues from the mucin structure [61,62]. Chitosan can form physical gels alone when an acidic solution of chitosan is exposed to an alkaline medium. It has been demonstrated that gelation time is dependent on the degree of deacetylation of chitosan. Compounds with a high degree of deacetylation have low density charge associated with the presence of numerous hydrophobic groups, which enable the gelling process [63]. Physically cross-linked gels are biocompatible and well tolerated due to the lack of chemical cross-linking agents. However, they do not have high mechanical stability and can react to environmental changes such as temperature, pH or ionic strength [64].

#### 2.3.1. Temperature-Triggered In Situ Gel Systems Based on Chitosan

Temperature-triggered in situ gel systems are liquid at room temperature (25 °C) and undergo a phase transition to the ocular surface due to physiological temperature (37 °C). The phase transition occurs when the thermosensitive polymer found in solution becomes insoluble above or below a certain temperature, called the lower critical solution temperature (LCST) and the upper critical solution temperature (UCST), respectively [65].

**Figure 7:**
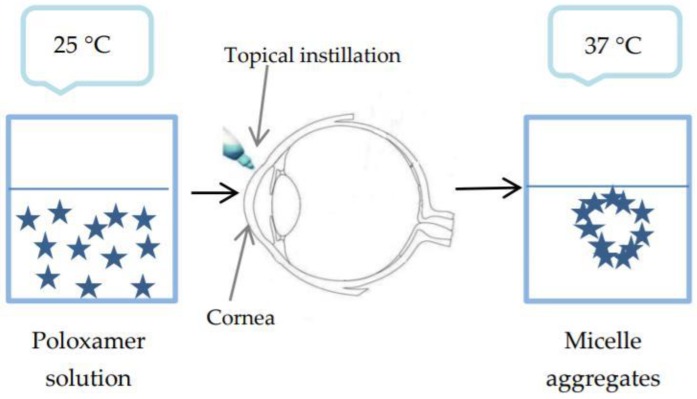
Sol-gel transition of poloxamer.

Poloxamers are thermosensitive polymers available in Europe under the name Lutrol^®^ and in the US. under the name Pluronics^®^. The most commonly used are Poloxamer 407 and Poloxamer 188 [66]. These are block copolymers made up of polyoxyethylene units and polyoxypropylene units that undergo a sol-gel transition when temperature rises. The gelation mechanism involves the formation of micelle aggregates dependent on temperature or polymer concentration (Figure 7) [67]. These spherical micelles have a dehydrated polyoxypropylene core and hydrated polyoxyethylene outer shell. Temperature rise causes dehydration and conformational changes in the hydrophobic chains. It also increases chain friction and entanglement of the polymeric network. More unbound water is available at hydrophilic regions, and polyoxyethylene chains interpenetrate the gel structure [27]. At concentrations of 18% or more in aqueous solution, poloxamer is converted from a low viscosity solution into a non-cross-linked gel at room temperature. If the preparation was stored in the refrigerator for easy administration, the low temperature of the preparation would produce a potential ocular irritation [68].

Chitosan solutions have positive effects on increasing the contact time with the ocular surface. Its association with poloxamer results in an enhanced mechanical strength of the formulations. For this purpose, Kadam et al. formulated in situ gels based on modified chitosan and poloxamer for ocular delivery of moxifloxacin hydrochloride. Poloxamer was used as a gelling agent, and an increase in its concentration lowered the phase transition temperature. The gelation temperature varied between 25 °C and 38 °C. Modified chitosan at different concentrations did not influence the gelation temperature, but enabled the formation of viscous solutions. The association with modified chitosan resulted in increased mucoadhesiveness and prolonged erosion time for the analyzed preparations [69].

Gels obtained by neutralizing chitosan with salts of polyols exhibit thermo-reversible gelling properties in aqueous medium. Such a strategy used the thermosensitive character of a physical mixture of chitosan and β-glycerophosphate (GP) or disodium β-glycerophosphate. The phosphate group in the glycerophosphate structure neutralizes the chitosan amino groups and increases the hydrophobic interactions and the number of hydrogen bonds between the chitosan chains at high temperature [29]. A thermosensitive ophthalmic gel that associates accuracy of dosing and ease of administration with bioavailability of gels was formulated by Fabiano et al. by gelling a chitosan hydrochloride solution with β-glycerophosphate in which 5-fluorouracil was incorporated. The authors of the study determined the sol-gel transition at 35 °C, the temperature of the corneal surface. They considered that that in situ gel increased ocular contact time and the bioavailability of 5-fluorouracil [70]. 

Derived from collagen, gelatin undergoes a reversible sol-gel transition at room temperature. It is a biocompatible polymer, but has low mechanical properties and therefore requires the addition of cross-linking agents [71]. A thermosensitive gel based on chitosan, gelatin and disodium β-glycerophosphate was developed as a delivery system for ferulic acid in corneal wound healing. The addition of β-glycerophosphate to the chitosan-gelatin mixture did not cause precipitation at pH 7.4 due to the neutralizing effect of GP. Chitosan and gelatin have hydrophilic functional groups capable of forming hydrogen bonds, which increase bioadhesion. These features determined the prolonged retention time of ferulic acid. The system was considered a promising approach in the treatment of corneal alkali burns [72]. Chemical cross-linking of chitosan leads to gels with a better mechanical strength compared to physical gels. The reaction between the primary amino group and an aldehyde forms a Schiff base. Among the cross-linking agents used is glutaraldehyde or genipin [73]. An in situ gel system with chitosan and gelatin co-cross-linked with β-glycerophosphate disodium salt hydrate (β-GD) and genipin was developed. Researchers observed that the polymeric mixture exhibited rapid gelation at 37 °C. Increasing gelatin concentration lowered gelation time due to the hydrogen bonds between the amino and hydroxyl groups of chitosan and the hydroxyl groups of gelatin [74].

Induced pluripotent stem cells (iPSCs) have a promising potential in corneal wound healing. To investigate the availability of iPSCs in corneal repair, Chien et al. developed a thermosensitive injectable hydrogel based on carboxymethyl hexanoyl chitosan (CHC) to enhance the viability of stem cells and to maintain gene expression in the culture medium. The gelation temperature was noted around 37 °C. The combination of iPSCs with that hydrogel favored healing of the cornea affected by surgical abrasion. In severe impairment, iPSC/CHC hydrogel enhanced corneal reconstruction by downregulation of oxidative stress and uptake of endogenous epithelial cells. The authors demonstrated that human keratocyte-reprogrammed iPSCs associated with the CHC gel represented a rapid and efficient delivery system in facilitating corneal wound healing [75].

#### 2.3.2. pH-Triggered In Situ Gel Systems Based on Chitosan

pH-sensitive polymers are compounds that have in their structure acidic (carboxylic or sulfonic) groups or basic (ammonium salts) groups that accept or release protons in response to changes in the pH of the medium.

**Figure 8:**
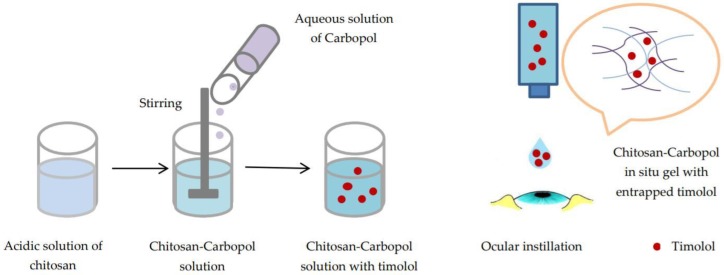
Chitosan-Carbopol in situ gel for ocular delivery of timolol.

One of the most commonly-used polymers with thixotropic properties is polyacrylic acid (PAA), whose aqueous solutions are acidic and less viscous [61]. Polyacrylic acid called Carbopol undergoes sol-gel transition once pH increases above 5.5. When pH rises, PAA swells due to electrostatic repulsions between negatively-charged carboxyl groups. Carbopol formulations have prolonged precorneal residence time due to their mucoadhesive properties being superior to other polymers [76]. Carbopol was associated with chitosan by Gupta et al. in in situ gels for ocular delivery of timolol maleate. The formulation containing 0.5% chitosan/0.4% Carbopol was a solution at room temperature and rapidly underwent a sol-gel transition due to the pH of the tear film (7.4) (Figure 8). Timolol was delivered for 24 h, following Fick’s law. According to the authors, the system was a viable alternative to conventional eye drops [77]. Chitosan and Carbopol have synergistic action regarding the mucoadhesive properties of the gels. Zaki et al. formulated an ophthalmic in situ gel based on chitosan and Carbopol 940 in which they incorporated ketorolac tromethamine. It displayed a better contact time with the ocular surface, increasing the healing rate of ocular ulcers in rabbits compared to conventional eye drops [78].

#### 2.3.3. Ion-Triggered In Situ Gel Systems Based on Chitosan

Ion-triggered in situ gels are systems made of polymers that undergo phase transition in the presence of electrolytes from the tear film. Gellan gum and sodium alginate are the most commonly-used ion-sensitive polymers in ophthalmic formulations [61].

**Figure 9:**
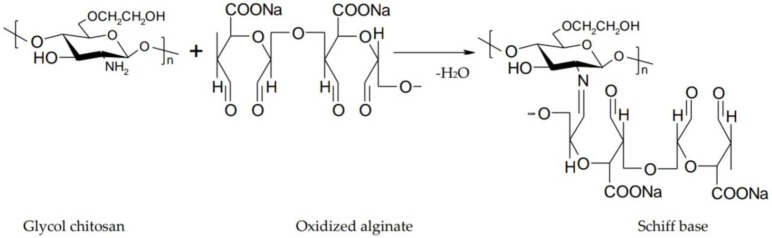
Interaction between glycol chitosan and oxidized alginate results in a Schiff base.

Alginate is a biocompatible and biodegradable anionic polysaccharide composed of β-d-mannuronic acid and α-l-guluronic acid residues linked by 1,4-glycosidic bonds. It increases ocular contact time due to its mucoadhesive character and gelling properties resulting from the interaction of guluronic acid residues with calcium ions in the tear fluid [65]. In order to improve mechanical properties and erosion resistance, it is associated with chitosan or its derivatives. Thus, the carboxyl groups of the alginate cause the formation of ionic bonds with the positive groups of chitosan with the appearance of a shell around the alginate gel, which becomes more resistant [79]. Gupta et al. formulated an in situ gelling system based on sodium alginate and chitosan for ocular delivery of levofloxacin, and they compared it with conventional ophthalmic drops. Chitosan turned into gel when the pH reached 7.4. Sodium alginate formed a gel upon contact with the divalent cations present in the simulated lacrimal fluid. Through a dual gelling mechanism, a gel with good stiffness and pseudoplastic behavior was obtained. Studies concluded that eye drops were rapidly removed from the corneal surface, while the in situ gel provided a prolonged retention time on the corneal surface [80]. Xu et al. developed an in situ gel based on glycol chitosan and oxidized alginate for ocular delivery of Avastin. Gelation was due to the interaction between the amino group of chitosan and the aldehyde group of alginate that results in a Schiff base (Figure 9). Glycol chitosan was used instead of chitosan because of its better solubility. By modulating the ratio between glycol chitosan and oxidized alginate, the gelation time ranged from 10 s–5 min [81].

Gellan gum is a natural anionic polysaccharide obtained from *Pseudomonas elodea*. It consists of α-l-rhamnose, β-d-glucuronic acid and d-glucose units. At room temperature, the polymer forms double helices in aqueous solution that are connected via van der Waals forces. Upon contact with cations of the lacrimal fluid, aggregation and cross-linking of the polymer occur [65]. Gellan gum has hydroxyl and carboxyl groups that can interact with other polymers by hydrogen bonds or electrostatic attraction [76]. Gupta et al. used chitosan, a pH-sensitive polymer and permeation enhancer, and gellan gum, an ion-sensitive polymer and a gelling agent, for development of in situ gels. According to the authors, the system consisting of 0.25% chitosan/0.5% gellan gum allowed easy instillation as eye drops that then underwent a sol-gel transition. The pH provides gelation of chitosan, and the ions of the tear fluid allowed the gelation of gellan gum. The advantages of this formulation compared to ophthalmic drops were enhanced transcorneal permeability and prolonged corneal retention time for timolol maleate [82].

## 3. Strategies to Increase Corneal Permeability

### 3.1. Permeation Enhancers

Increasing drug permeability through corneal epithelium can be achieved by using permeation enhancers that transiently decrease corneal barrier resistance. The addition of penetration enhancers to an ophthalmic solution reduces the size of the instilled drop and improves the bioavailability of poorly absorbable substances. Most substances are surfactants that alter the physical properties of cell membranes by removal of phospholipids or membrane solubilization. Benzalkonium chloride is a conventional permeation enhancer that alters the ocular barrier, but can lead to toxic effects by accumulation in the cornea for several days. The mechanism of action of ethylenediaminetetraacetic (EDTA) is to alter tight junctions between superficial epithelial cells and to facilitate paracellular transport [83,84].

Numerous studies have demonstrated the potential of chitosan as a permeation enhancer for the absorption of hydrophilic drugs. Chitosan is able to adhere to the surface of the mucosa and temporarily open tight junctions between cells [85]. Majumdar et al. evaluated the effect of chitosan, benzalkonium chloride (BAK) and disodium ethylenediaminetetraacetic acid (EDTA), alone and in combination, on acyclovir permeability through the rabbit excised cornea. The authors observed that 0.1% chitosan alone increased acyclovir permeability by 3.1 times, while chitosan at the same concentration in the presence of the other two promoters generated a 5.5-fold increase. The conclusion of the study was that the association of the three compounds resulted in a significant increase in corneal permeability of acyclovir and that chitosan improved the diffusion of hydrophilic compounds through the corneal membrane [86].

Cyclodextrins solubilize lipophilic compounds by complexation and lead to enhanced drug permeability through the eye with a good bioavailability of the active substance. To achieve optimal bioavailability, concentrations of 15% or less should be added to an aqueous ophthalmic solution. Addition of large amounts decreases drug bioavailability by retaining active molecules in the tear film [83]. Mahmoud et al. developed nanoparticles of chitosan and sulfobutyl ether-β-cyclodextrin (SBE-β-CD) for sustained release of econazole nitrate in the eye. The use of low molecular weight chitosan causes the formation of nanoparticles with a large surface area. Low molecular weight chitosan formulations have low viscosity and the ability to form smaller structures. The results showed that nanoparticle with econazole provided an increased antifungal effect compared to a solution of econazole [87]. In a similar manner, Zhang et al. prepared sulfobutyl ether-β-cyclodextrin/chitosan nanoparticles as a potential ophthalmic delivery system for naringenin. In vivo studies suggested that nanoparticles were non-irritating to the rabbit eye and had a superior bioavailability of naringenin in aqueous humor compared to naringenin suspension. The authors concluded that nanoparticles based on sulfobutyl ether-β-cyclodextrin/chitosan were an alternative for ocular administration of poorly-soluble substances [88].

**Figure 10:**
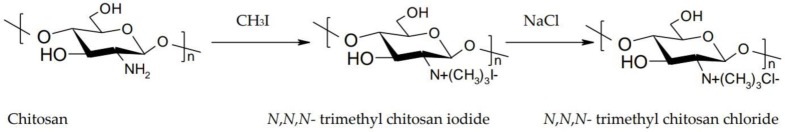
Schematic illustration of *N*-trimethyl chitosan synthesis.

Due to the presence of functional groups, chitosan is a versatile polymer that allows the synthesis of derivatives with multifunctional properties. Chitosan derivatives seem to enhance the paracellular transport of drugs by opening tight junctions. The mucoadhesive properties of chitosans give additional value to the development of transmucosal drug delivery systems. Nanosystems based on chitosan or its derivatives increase interaction with mucous membranes, facilitate the transport of macromolecules and protect proteins. The most plausible hypothesis of penetration enhancement is endocytosis [89]. The use of unmodified chitosan as a corneal penetration enhancer encounters the problem of low solubility in the lacrimal fluid at physiological pH. Polycyclic derivatives of chitosan are soluble in the tear film at physiological pH and have a high potential for increasing corneal permeability. The *N*-trimethyl chitosan derivative, known as TMC, is obtained by quaternization of the primary amino group of chitosan with methyl iodide (Figure 10). The efficiency of enhancing the permeability depends on the charge density, which, in neutral or alkaline medium, is determined by the degree of quaternization [90]. TMC with a 60% trimethylation degree promotes drug permeability through cell monolayers, demonstrating that a threshold value of polymer charge density is required to open tight junctions at neutral pH [89].

Nanoparticles of *N*-trimethyl chitosan were prepared to improve the ocular bioavailability of sodium diclofenac. For this purpose, TMC with a quaternization degree of 49.8% was used. Nanoparticles showed a prolonged time to reach the maximum concentration of diclofenac in aqueous humor. The nanoparticles determined an increased absorption of diclofenac due to mucoadhesive and absorption enhancer properties of TMC [91]. Di Colo et al. prepared TMC polymers with different quaternization degrees and molecular weights. The derivatives were compared regarding the ability of increasing the permeability of ofloxacin through the rabbit corneal epithelium. Polymers with an intermediate quaternization degree significantly enhanced permeability, independently of molecular weight. A possible explanation would be that at an intermediate degree of quaternization, the TMC structure is flexible. At high quaternization degrees, electrostatic interactions between TMC and epithelial cells are hindered by the steric effects of attached methyl groups [92].

Chitosan toxicity is dependent on the degree of deacetylation (DD) and molecular weight (Mw). At high DD, toxicity is Mw and concentration dependent, and at low DD, toxicity is less pronounced. Trimethyl chitosan is a compound with relatively low toxicity, but a high trimethylation degree enhances cytotoxicity. This is related to the density of the molecule’s charge. It is a threshold level below which there are few contact points between the polymer and cell components to produce a significant toxic effect. The balance is between 40% and 60% DD or degree of trimethylation, although any chitosan with Mw < 10 kDa is not considered to be toxic. Thus, changes that do not increase the charge of the molecule appear to have little effect on cellular toxicity [93].

Quaternization of chitosan leads not only to the increase of cell solubility and permeability, but also to the toxicity manifested by the production of reactive oxygen species and the inhibition of cellular proliferation. A study by Zubareva et al. evaluated the cytotoxicity of chitosan and chitosan derivatives using the MTT assay. Unmodified chitosan, as well as the quaternary derivative with substitution degree 9% (CHq9) slightly decreased cell viability, while the derivative with substitution degree 98% (CHq98) was toxic in a wide range of pH. Unmodified chitosan, CHq9 and the hydrophobic derivative CHq 88h10 (hexanoyl derivative) had no effect on the cell cycle. CHq98 interrupted cell division in the G2/M phase. Modifying these derivatives with hydrophobic residues has diminished cytotoxic activity while maintaining cell penetration [94].

**Figure 11:**
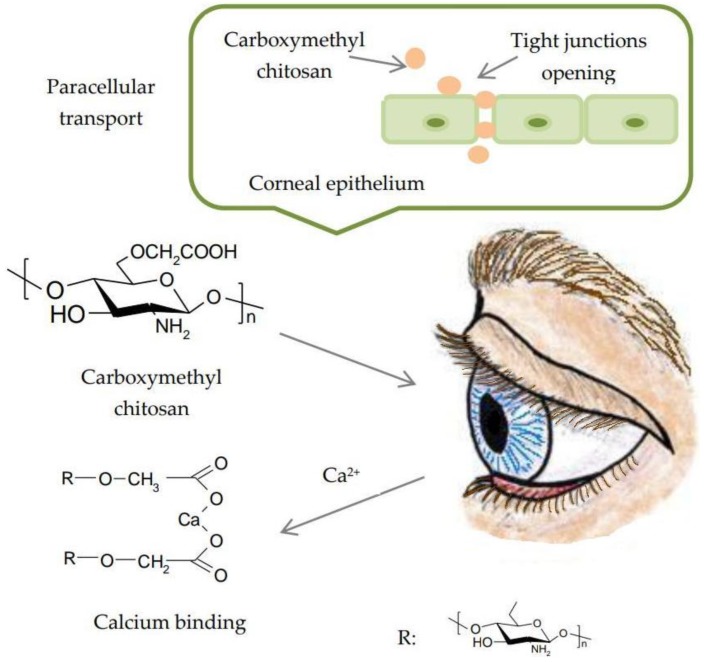
Paracellular transport of carboxymethyl chitosan through the corneal epithelium. Reproduced with permission from Ref. [84]. Copyright © Elsevier, 2014.

Substitution with alkyl or carboxymethyl groups increases solubility. The reactive sites for chemical modifications are the amino group at C2 and hydroxyl groups at the C3 and C6 positions. Positions C2 and C6 are more susceptible to substitution. O-carboxymethyl chitosan has an increased capacity of binding calcium due to the presence of a carboxymethyl group, depriving the extracellular matrix from calcium ions. Thus, it increases paracellular permeability through the epithelium (Figure 11) [95]. 

The mechanism responsible for the higher permeability of thiolated derivatives is the inhibition of tyrosine phosphatase involved in the closure and opening of tight junctions. Tyrosine phosphatase determines the dephosphorylation of tyrosine subunits from the constitution of occludin, a transmembrane protein from tight junctions. When these subunits are dephosphorylated, the junctions are closed [46]. Schuerer et al. evaluated the effect of chitosan and chitosan-*N*-acetylcysteine (CS-NAC) on conjunctival epithelial cells. Unlike chitosan-*N*-acetylcysteine, chitosan rapidly permeabilized conjunctival epithelial cells in vitro. In vivo studies were performed on guinea pigs. The structural and functional similarities between guinea pigs and humans make data obtained from guinea pigs the most valuable in predicting local immune response and diseases in humans. According to the immunohistological analysis, chitosan penetrated the tissue up to 12 min after instillation, and it was detected in conjunctival epithelial cells. Fluorescent signals were detected not only in the outer layer of the conjunctival epithelium, but also in cells at about 80 μm. This fact suggested that the permeability of chitosan in connective tissue is mediated via intracellular and paracellular pathways. Measurements of the transepithelial electrical resistance assay evaluated the effect of chitosan on the barrier function of conjunctival epithelial cells. This indicated the opening of tight junctions. For 24 h, cells were unable to restore tight junctions once opened [96]. CS-NAC has different characteristics that recommend it as a candidate in ophthalmic formulations. Liu et al. investigated whether nanostructured lipid carriers (NLC) coated with chitosan-*N*-acetylcysteine could increase precorneal retention time and corneal permeability of curcumin. Following the ex vivo cornea penetration study, penetration curves were linear, which confirmed the maintenance of corneal integrity during the experiment. The preparations released curcumin following zero-order kinetics, and the reduction in the corneal permeability rate was observed at 60 min. One possible explanation would be that for 60 min, curcumin passed through the corneal epithelium to the hydrophilic stroma, where it formed a reservoir. In addition, CS-NAC enabled the opening of tight junctions by inhibiting tyrosine phosphatase protein and enhancing paracellular transport of curcumin. It also had mucoadhesive properties by forming disulfide bonds with cysteine residues in mucus. In conclusion, chitosan-*N*-acetylcysteine-based NLC increases ocular curcumin bioavailability by enhancing transcorneal permeability and ocular contact time [97]. Further, nanostructured lipid carriers (NLCs) based on chitosan derivatives such as CS-NAC, chitosan oligosaccharides (COS) and carboxymethyl chitosan (CMCS) were developed. In vitro corneal permeation studies showed that COS and CS-NAC-coated NLCs penetrated through the corneal epithelium barrier (about 40 μm), while CMCS failed to enhance intraocular drug penetration as expected, displaying a negligible fluorescence at 30 μm deep. According to the authors, CS-NAC-NLC and COS-NLC were promising ocular drug delivery systems in order to achieve prolonged precorneal retention, higher corneal permeability and enhanced ocular bioavailability [98].

### 3.2. Prodrugs

Prodrugs are pharmacologically inactive compounds and derivatives of molecules that require chemical or enzymatic transformation for the release of the active substance. Enzymatic transformation of prodrugs into ophthalmic tissues is used to release the parent drug. Active substances possess hydroxyl or carboxyl groups, which can be esterified to produce lipophilic compounds. The activity of esterase is 2.5-fold higher in the corneal epithelium compared to stroma and endothelium. Absence of acetylcholinesterase and butylcholinesterase in tears allows the absorption of the prodrug through the corneal epithelium. Incorporation of prodrugs into various delivery systems associates an enhanced permeability of the active substance through the cornea with prolonged precorneal retention [99].

Ganciclovir is an antiviral compound that has significant activity on human cytomegalovirus. The partition coefficient is low, so that the ocular bioavailability is poor [100]. Thus, Kapanigowda et al. incorporated ganciclovir into chitosan microspheres. Polymeric microspheres had the advantages of easy administration in liquid form and rapid diffusion in ocular tissues. The polymeric matrix of chitosan facilitated diffusion of the microspheres. A positive zeta potential enabled adhesion to the surface of the cornea and prevented nasolacrimal drainage. Microspheres interacted with the cell membrane, resulting in a structural reorganization of proteins of tight junctions. Thus, transcorneal permeability was achieved. The concentration of ganciclovir in the aqueous humor of Wistar rats after administration of the microspheres was significantly higher than that obtained from the instillation of a solution [101].

Prostaglandin analogues are carboxylic acids with low permeability, used as hypotensive agents. These carboxyl groups are used for the synthesis of alkyl/aryl ester prodrugs with increased lipophilicity and transcorneal permeability. They have more potent hypotensive action than the parent drug. Because of the irritant effect of prostaglandin analogs, prostaglandin derivatives such as latanoprost exhibit elevated selectivity for the prostaglandin F receptor. Latanoprost undergoes hydrolysis under the action of corneal esterase at biologically-active latanoprost. The cornea slowly releases latanoprost acid to the anterior segment of the eye [100]. Cheng et al. developed a thermosensitive injectable hydrogel based on chitosan, gelatin and glycerol phosphate as a sustained release system of latanoprost in the treatment of glaucoma. After the sub-conjunctival injection of hydrogel in the rabbit model, the intraocular pressure lowered within eight days and remained normal for 31 days. The results of the in vivo tests showed a steady state concentration of the drug in the aqueous humor without burst release [102].

With regard to beta blockers, a typical prodrug example is timolol. At physiological pH, 98% of timolol is protonated and has low lipophilicity. The hydrophilic nature reduces corneal permeability, and less than 5% is absorbed into the internal structures of the eye [99]. Zhao et al. formulated nanoparticles based on galactosylated chitosan. They carried out the potential of the ophthalmic delivery of timolol maleate with the purpose of enhanced therapeutic effects and diminished side effects. Compared to conventional eye drops, nanoparticles exhibited a permeability coefficient of 1.45-fold greater. This was related to an increased oil-water partition coefficient and nanoparticle size. High lipophilicity enhanced the permeability of the corneal epithelium. Additionally, the positive charge of nanoparticles allowed interaction with mucin from the ocular surface [103].

Nepafenac is an amide prodrug, used in ophthalmic formulations for the treatment of pain and inflammation associated with cataract surgery [104]. The purpose of a study initiated by Yu et al. was the incorporation of nanostructured lipid carriers (NLC) with nepafenac into a thermosensitive gel of poloxamer and carboxymethyl chitosan to enhance the transcorneal permeability of the active substance. The transcorneal penetration was evaluated using isolated rabbit corneas, with a significantly high apparent permeability coefficient of 2.36-times that of eye drops. This could be attributed to the fact that the lipid vesicles were biocompatible with the corneal epithelial cells, leading to increased solubility and easy transport through the cornea of encapsulated drug. Meanwhile, the NLC contains surfactants, which opens the tight junctions in the epithelial cornea and facilitates the corneal permeation of the drug [105].

### 3.3. Colloidal Systems: Nanoparticles and Liposomes

Colloidal systems are likely to provide a controlled drug release with prolonged pharmacological effects. Such an objective can be achieved by a retention localized in the cul-de-sac where the entrapped drug can be delivered by diffusion or under external stimuli such as light. These formulations may have prolonged contact with the ocular surface, withstanding ocular clearance mechanisms. To achieve the optimal effect, it is necessary to create mucoadhesive nanosystems in order to enhance ocular bioavailability [106]. Different types of chitosan-based nanosystems have an increased ability to improve ocular drug delivery. Hydrophilic and hydrophobic drugs, as well as biomacromolecules can be delivered after inclusion in chitosan-based colloidal systems. Depending on whether chitosan is in the form of a coating or a nanomatrix, nanocarriers can be chitosan-coated or chitosan-based nanosystems [107].

Nanoparticles have a diameter of less than 1 μm and are made up of natural or synthetic polymers in which the drug is dissolved, incorporated or encapsulated. Drugs can also be integrated into the matrix or surface. Nanoparticles are well tolerated by patients [83]. Nanoparticles have been designed to overcome ocular barriers, increase drug permeability and maintain optimal concentration of active substances in target tissues [108].

While causing prolonged ophthalmic release of active substances, they may have a number of side effects such as tissue accumulation, blockage of punctal drainage by aggregation or damage to lacrimal fluid recycling [83]. The features of chitosan nanoparticles for ocular administration include mild preparation conditions, the ability to obtain homogeneous particle populations and the modulation of surface size and surface charge, the possibility of associating different types of compounds such as proteins or nucleic acids and the incorporation of molecules in the nanomatrix structure [107]. Some examples of chitosan nanoparticles are summarized in Table 1.

Liposomes are small spherical vesicles consisting of cholesterol and natural phospholipids. Due to their biocompatibility, biodegradability, non-toxicity and ability to incorporate lipophilic and hydrophilic compounds, liposomes are promising drug delivery systems [120]. Topical administration of chitosan-coated liposomes (chitosomes) improves precorneal retention and slows down drug metabolism to the ocular surface. In addition, degradation of chitosan to oligosaccharides under the action of lysozyme leads to non-toxic compounds [121]. High molecular weight chitosan-coated liposomes have a smaller size due to the complete coating of the liposome surface, which acts as a physical barrier that inhibits aggregation. Liposomal surface charge influences particle size. Negatively-charged liposomes have a larger diameter because there are electrostatic attractions between positively-charged chitosan and negatively-charged phospholipids. In addition, chitosomes have a slow drug release rate compared to uncoated liposomes due to the additional barrier that decreases drug diffusion [122]. Some examples of liposomes coated with chitosan are shown in Table 2.

## 4. Conclusions

Overcoming the inconvenience of conventional eye drops is a difficult task because the eye is a sensitive organ and the formulations must be safe and efficient without disturbing the vision process. In order to increase drug bioavailability, two strategies have been developed that involve increasing ocular contact time and enhancing corneal permeability. Chitosan is a versatile polymer used in ophthalmology due to properties such as biocompatibility, biodegradability, mucoadhesive character and antimicrobial activity. Chitosan plays a key role in improving corneal wound healing by stimulating the migration of keratinocytes, which leads to rapid growth of collagen production. Used alone or in combination with other compounds with synergistic action, chitosan increases ocular retention time and corneal permeability. The presence of the amino group and the cationic behavior in acidic solutions explain the unique properties among other biopolymers. Chitosan-based drug delivery systems such as in situ gels, nanoparticles or liposomes provide ease of administration, protection for entrapped drugs, rapid release, increased ocular retention time and bioavailability of the active substances.

Nowadays, most chitosan-based systems are designed for the ocular delivery of a single therapeutic agent, so in the future, they could be improved by associating active substances with synergistic action to enhance the therapeutic effect in the treatment of ocular disorders. 

The eye is a sensitive organ, and the viscosity of a gel can create discomfort for certain patients. Rigorous viscosity control in the formulation process is necessary to avoid such inconvenience. Nanoparticles can cause blockage of punctal drainage, so further studies are necessary to assess the risks of prolonged and repeated administration of these pharmaceutical forms. 

Current studies show that chitosan is a non-toxic and biocompatible polymer. However, factors such as deacetylation degree or molecular weight may influence the toxicity of chitosan. In-depth studies for in vivo and in vitro toxicity are required to correlate the structure of chitosan and its derivatives with their safety profile.

## Figures and Tables

**Table 1:** Chitosan-based nanoparticles for ocular drug delivery.

Drug	Main Excipient(s)	Major Findings	Clinical Indications	Ref.
Daptomycin	Chitosan and sodium tripolyphosphate (TPP) nanoparticles	This nanoparticulate system could arise as a possible way to deliver the antibiotic directly to the site of action and enhance its residence time in the eye.	Bacterial endophthalmitis	[109]
Indomethacin	Chitosan and sodium tripolyphosphate (TPP) nanoparticles and nanoemulsion	In vivo studies and histopathological examination revealed that rabbits’ eyes treated with nanoemulsion showed healing of corneal chemical ulcer with moderate inhibition of polymorph nuclear leukocytic infiltration (PMNLs) compared with nanoparticles.	Post-operative inflammation, healing of corneal ulcers	[110]
Moxifloxacin	Chitosan-dextran sulfate nanoparticles	Formulation exhibited biphasic release profile with an initial fast release followed by sustained release in next 24 h. Moxifloxacin loaded nanoparticles exhibited a higher transcorneal permeation as well as significantly higher corneal retention compared to solution.	Ocular infections	[111]
Plasmid DNA	Hyaluronic acid-chitosan oligomer nanoparticles (HA-CSO NPs)	HA-CSO NPs had no effect on cell viability. The transfection efficiency of the model plasmid was significantly higher in NP treated cells than in controls.	Ocular surface disorders	[112]
Trichostatin A, Dominant negative survivin protein (SurR9-C84A)	Ultra-small chitosan nanoparticles (USC-NPs)	A combination of TSA with SurR9-C8A worked in synergy and showed a promising healing and anti-inflammatory effect in alkali burnt cornea.	Corneal wound healing	[113]
Bromfenac sodium	Chondroitin sulfate (ChS)-chitosan (CS)-nanoparticles (NPs)	Significantly high transcorneal permeation (1.62-fold) and corneal retention (1.92-fold) of bromfenac was observed through ChS-CS-NPs when compared with marketed eye drops.	Ocular inflammation	[114]
Cyclosporine A	Nanoparticles containing three types of chitosan with different molecular weights	CsA could be detected in both aqueous and vitreous humor samples up to 72 h. In vivo release profiles indicated prolonged release of active agent from nanoparticles containing chitosan with medium molecular weight.	Ocular inflammatory diseases	[115]
Carteolol	Chitosan nanoparticles (CS-NP)	In vitro release studies displayed a sustained release for 24 h as compared to drug solution. Ex vivo studies showed good permeation and safe nature for NP.	Glaucoma	[116]
Bovine lactoferrin (bLf)	Ultra-small algal chitosan nanoparticles (US CS NPs)	The in vivo and ex vivo biodistribution results suggested that the ultra-small CS NPs efficiently internalized into the ocular tissues within 1 h after administration. Ultra-small algal nanocarriers with bLf protein exhibited potential in inhibiting carbendazim-induced human lens cell apoptosis and oxidative stress.	To prevent carbendazim-induced toxicity	[117]
Betaxolol hydrochloride	Chitosan nanoparticles	The in vitro release studies in simulated tear fluid exhibited biphasic release pattern with an initial burst followed by sustained release up to 12 h. The developed nanoparticles showed significant decrease in intraocular pressure (IOP) compared to marketed formulation.	Glaucoma	[118]
Levofloxacin	Chitosan nanoparticles	Hen’s egg-chorioallantoic membrane test (HET-CAM test) and histopathology of cornea demonstrated that the formulation was non-irritant and safe for ocular administration. The antimicrobial study revealed higher antibacterial activity against *P. aeruginosa*, and *S. aureus*.	Ocular infections	[119]

**Table 2:** Chitosan-based liposomes for ocular drug delivery.

Drug	Main Excipient(s)	Major Findings	Clinical Indication	Ref.
Cyclosporine A (CsA)	Low molecular weight chitosan coated liposomes (LCHL)	In vitro drug release measurement showed that LCHL had a delayed release profile compared with non-coated liposomes. In vivo study in rabbits showed that the concentrations of CsA in cornea, conjunctiva, and sclera were remarkably increased by LCHL.	Ocular inflammatory diseases	[123]
Bromfenac (BRF)	Chitosan-coated liposomes	Release of BRF from liposomes was sustained for several hours depending on lipid concentration, inner water phase, initial drug amounts and surface properties.	Retinal and choroidal neovascularization, cystoid macular edema	[124]
Ciprofloxacin hydrochloride (CPX)	Chitosan-coated liposomes	Results showed an alteration in release rate and encapsulation efficiency of CPX from liposomal formulae upon varying the molar ratios of the lipid bilayer composition.	Ocular infections	[125]
Flurbiprofen (FP)	Chitosan-coated deformable liposomes (DL-CS)	The apparent permeability coefficient of FP-DL-0.1% CS evaluated using isolated rabbit corneas was 1.29-, 1.95- and 4.59-fold greater than that of uncoated FP-DL, conventional liposomes and FP solution.	Ocular inflammations	[126]
Timolol maleate ™	Chitosan coated liposomes (TM-CHL)	The TM-CHL exhibited significant mucin adhesion compared to commercial eye drops. TM-CHL produced a 3.18-fold increase in the apparent permeability coefficient resulting in a significant enhancement of corneal permeation.	Glaucoma	[127]
Coenzyme Q10	Trimethyl chitosan (TMC)-coated liposomes	A 4.8-fold increase in the precorneal residence time was achieved in the presence of TMC with a higher Mw compared with the control solution. The Draize test demonstrated the excellent ocular tolerance of TMC for topical administration.	Selenite-induced cataract	[128]
Diclofenac sodium	Low molecular weight chitosan (LCH)-coated liposomes	The LCH coating displayed a potential penetration enhancing effect for transcorneal drug delivery. In the ocular tolerance study, no irritation or toxicity was observed by continual administration of LCH- coated liposome in 7 days.	Ocular inflammatory diseases	[129]
Curcumin	Thiol derivatized chitosan (CSSH) coated liposomes	The CSSH coated curcumin liposomes (Cur-Lip-CSSH) showed slower in vitro release than Cur-Lip at pH 5.5 and pH 7.4. Treatment of MCF-7 cells with curcumin and Cur-Lip-CSSH showed dose and time dependent cytotoxicity.	Posterior ocular diseases	[130]
